# Research Trends in Evidence-Based Medicine: A Joinpoint Regression Analysis of More than 50 Years of Publication Data

**DOI:** 10.1371/journal.pone.0121054

**Published:** 2015-04-07

**Authors:** Bui The Hung, Nguyen Phuoc Long, Le Phi Hung, Nguyen Thien Luan, Nguyen Hoang Anh, Tran Diem Nghi, Mai Van Hieu, Nguyen Thi Huyen Trang, Herizo Fabien Rafidinarivo, Nguyen Ky Anh, David Hawkes, Nguyen Tien Huy, Kenji Hirayama

**Affiliations:** 1 University of Medicine and Pharmacy, Ho Chi Minh City, Ho Chi Minh, 70000, Vietnam; 2 Online Research Club, http://onlineresearchclub.org; 3 School of Medicine, Vietnam National University, Ho Chi Minh City, Ho Chi Minh, 70000, Vietnam; 4 University of Medicine and Pharmacy at Hue City, Hue, 53000, Vietnam; 5 Nagasaki University Graduate School of Pharmaceutical Sciences, Nagasaki, 852–8523, Japan; 6 The Florey Institute of Neuroscience and Mental Health, The University of Melbourne, Victoria, 3010, Australia; 7 Department of Clinical Product Development, Institute of Tropical Medicine (NEKKEN), Nagasaki University, 1-12-4 Sakamoto, Nagasaki, 852–8523, Japan; 8 Department of Immunogenetics, Institute of Tropical Medicine (NEKKEN), Nagasaki University, 1-12-4 Sakamoto, Nagasaki, 852–8523, Japan; Middlesex University Dubai, UNITED ARAB EMIRATES

## Abstract

**Background:**

Evidence-based medicine (EBM) has developed as the dominant paradigm of assessment of evidence that is used in clinical practice. Since its development, EBM has been applied to integrate the best available research into diagnosis and treatment with the purpose of improving patient care. In the EBM era, a hierarchy of evidence has been proposed, including various types of research methods, such as meta-analysis (MA), systematic review (SRV), randomized controlled trial (RCT), case report (CR), practice guideline (PGL), and so on. Although there are numerous studies examining the impact and importance of specific cases of EBM in clinical practice, there is a lack of research quantitatively measuring publication trends in the growth and development of EBM. Therefore, a bibliometric analysis was constructed to determine the scientific productivity of EBM research over decades.

**Methods:**

NCBI PubMed database was used to search, retrieve and classify publications according to research method and year of publication. Joinpoint regression analysis was undertaken to analyze trends in research productivity and the prevalence of individual research methods.

**Findings:**

Analysis indicates that MA and SRV, which are classified as the highest ranking of evidence in the EBM, accounted for a relatively small but auspicious number of publications. For most research methods, the annual percent change (APC) indicates a consistent increase in publication frequency. MA, SRV and RCT show the highest rate of publication growth in the past twenty years. Only controlled clinical trials (CCT) shows a non-significant reduction in publications over the past ten years.

**Conclusions:**

Higher quality research methods, such as MA, SRV and RCT, are showing continuous publication growth, which suggests an acknowledgement of the value of these methods. This study provides the first quantitative assessment of research method publication trends in EBM.

## Introduction

From the 1900s until now, evidence-based medicine (EBM) has developed into the dominant paradigm for clinical practice [1–3]. Although the term EBM officially appeared for the first time in 1992 in an article by Guyatt et al in JAMA [4], traces of the origins of EBM dated back to ancient Greece [5,6]. By 1996, EBM was formally defined as “the conscientious, explicit and judicious use of current best evidence in making decisions about the care of individual patients” by Sacket et al [7] and this definition has been recognized and strongly endorsed by most of the world's scholarly articles on EBM [8–10]. It is important to note that while often used interchangeably, EBM and science-based medicine (SBM) are related but different terms. SBM is a subset of EBM which not only involves evidence for treatment efficacy but also a mechanism by which the effect can occur. One (historical) example of a treatment that is EBM but not SBM is a number of different forms of anaesthetic which have been clearly shown to work but the mechanism remains unclear [11]. Internationally, EBM now provides the framework for the diagnosis and treatment of most health conditions [12–14]. The alternative to EBM is empirical diagnosis and treatment, which is a system much more open to individual, cultural and training bias [15]. Overall this approach has become less popular as health practitioners have greater access to cutting edge medical knowledge in the current information era. The increasing rate of research and knowledge acquisition often means that clinicians are asked questions the answers to which have changed since their training [16]. Patients expect physicians to be able to interpret and explain medical information from a wide range of sources including the internet [11,17]. Insurers expect physicians to know which diagnostic and treatment strategies strike the best balance between accuracy and cost effectiveness [18]. While students need to rapidly assess medical information and its quality, they must also learn to make decisions in the absence of good evidence [19]. EBM provides a framework for using medical and scientific evidence to effectively guide clinical practice, and as such is thoroughly prepared to match all of these challenges [4,12,19–21].

The basic principle of EBM is simply that we should treat when the evidence indicates that perceived benefits outweigh the perceived risks and conversely not treat when the risks are higher than the benefits. Assessments using EBM have to be conducted in 5 key steps: defining the clinical question, finding the best evidence, critically appraising the evidence, applying the evidence to the patient and evaluating the performance of the decision [22,23]. Finally, the evidence should be presented and assessed through a logical and systematic classification in which the value of evidence can be ranked [24]. This system allows assessment of the quality of studies and often informs recommendation for changes in best clinical practice [25].

There is no single, universally-accepted hierarchy of evidence [26]. Yet most people agree that current, well-designed systematic reviews (SRV) and meta-analyses (MA) are at the least risk of bias and hence represent the most robust, high quality evidence while case reports or expert opinions are considered having the highest risk of bias [27–31]. Other methods such as randomized controlled trials or cohort studies fit in somewhere in the middle in terms of research bias [29–33].

Despite its critical role in medical teaching, research, and clinical practice, there is a dearth of literature measuring the interest of researchers in EBM. The contribution of published research focusing on EBM over time has not been examined. Moreover, any changes in the proportions of the various study methods in the EBM hierarchy remain unclarified. The current study involved a bibliometric investigation to evaluate trends in research productivity and the contribution of different research methods to EBM. This study used the US National Library of Medicine’s PubMed database to find articles published over a period of 68 years (1945–2012) sorted by journal of publication, taking advantage of the fact that PubMed facilitates filtering by article type. This study allows quantitative assessment of the issues outlined above and highlights significant trends in EBM research publication.

## Method

### Data collection

In this study, the filter tool available as part of PubMed (http://www.ncbi.nlm.nih.gov/PubMed) was used to search and classify all publications according to their article types with the following strategy: typing “all [sb]” in the search field, we initially searched All PubMed publication (APP). Searches were then limited by selecting only one of these article types: Case Report (CR), Clinical Trial (CT), Controlled Clinical Trial (CCT), Randomized Controlled Trial (RCT), Guideline (GL), Practical Guideline (PGL), Systemic Review (SRV) or Meta-Analysis (MA) in the filter tool. Hence, the annual number of publications of APP, CR, CT, CCT, RCT, GL, PGL, SRV and MA were retrieved regardless of text attainability, study design, publication date, language or species. Other types of publications, which account for up to 86.94% of APP, are neither well categorized by PubMed nor included in the hierarchical system of classifying evidence, and therefore are not included in this research. The level of evidence is varied and depends on many factors, e.g. the area that being researched, study quality, size of study population, etc… [34] However, the article types which are chosen for this study are arranged, from high to low weight of evidence according to general hierarchical order: MA, SRV, RCT, CCT, CR [28]. The Oxford Centre for Evidence-based Medicine (OCEBM) does not mention PGL and GL, but in other classification, they might place as the highest rank of evidence [34–36]. Review articles were not included in this strategy because the variability within this category would not allow non-clinical publications to be excluded. In this study, only publications from completed years were included, as a result, all publications after 2012, which are still being updated, were excluded. In each category, articles which did not form part of a single continuous series of annual data points and jump-shift count possibly due to categorization changes, as happened in cases of CR (before 1977) and CT (before 1961), were excluded. Hence, the PubMed database search identified bibliographic details in the following time periods: 1977–2012 for CR, 1961–2012 for CT, 1966–2012 for CCT, 1966–2012 for RCT, 1973–2012 for GL, 1978–2012 for PGL, 1945–2012 for SRV, 1990–2012 for MA, and 1945–2012 for APP (as that is the earliest and latest date for the subgroup categories).

### Data Analysis

All data extracted were analyzed using the Joinpoint Regression Program version 4.1.0 (Statistical Research and Applications Branch, National Cancer Institute, USA) [37] to examine the trends, and assess the significance of changes in trends, in the various study methodologies in the EBM hierarchy. Joinpoint regression was performed to identify periods with statistically distinct log-linear trends in number of publication of each article type over time [38–41]. The analyses determined the joinpoints at which there is an essential change in the trends with Bonferroni adjustment [42,43]. The detailed pattern of this model was first introduced and fully established by Kim HJ and colleagues [40].

**Fig 1:**
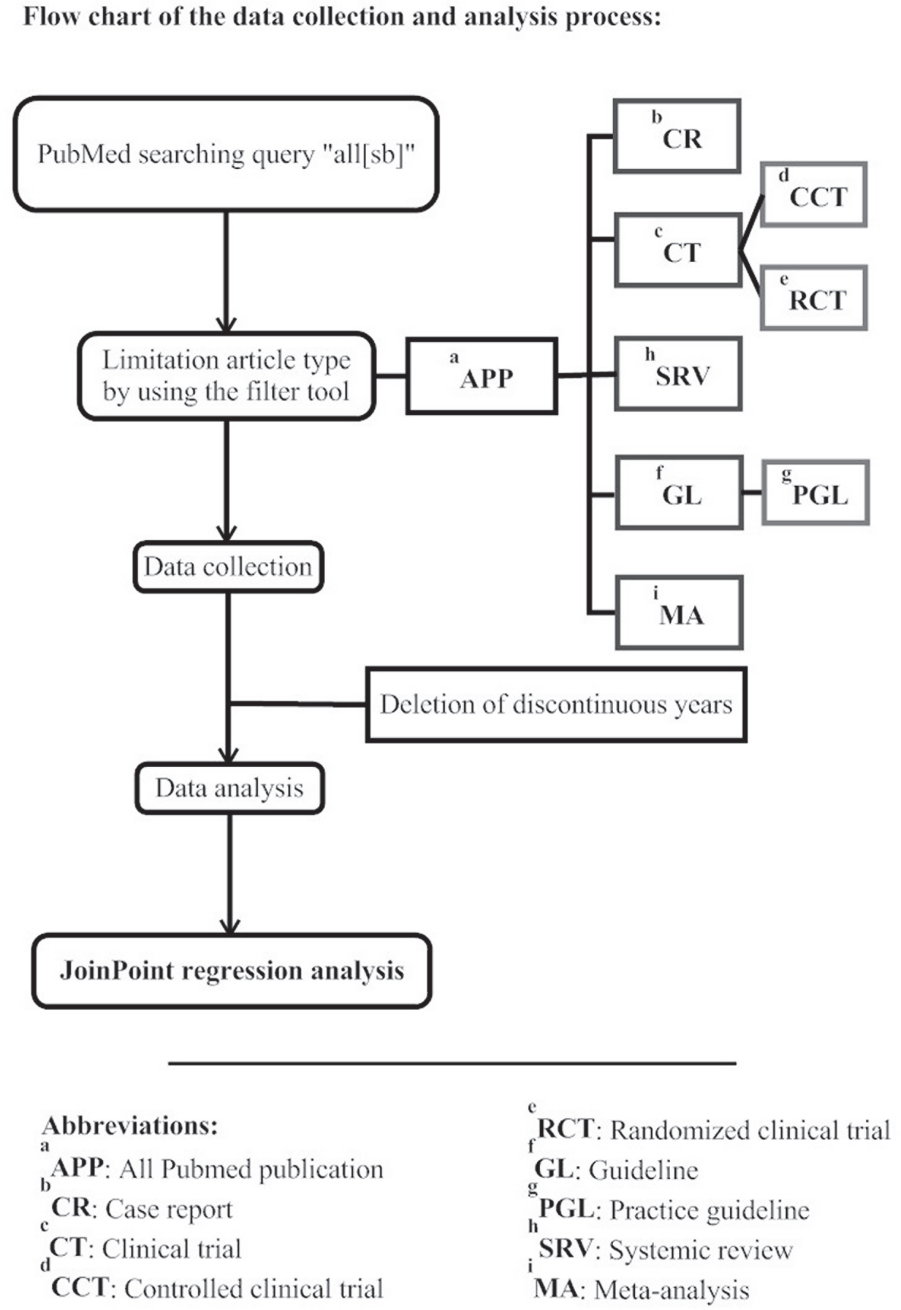
Flow chart of the data collection and analysis process.

We assigned the year of publication as an independent variable and the annual number of publications in every category and relative publication number as dependent variables for each Joinpoint session. Within the Data File Import Wizard, we established the Delimiter box as “Comma”, Missing Characters box as “Space”, Dependent Variable Information as “Provided”. The number of publications (in categories such as CR, CT…) was set as “Count” whilst proportion in each article type (such as the proportion of CT to APP) was set as “Proportion”. In the Specifications tab, “Shift data points” was set as “0”, “Number of Joinpoints” ranged from “0” to “3”, “Heteroscedastic Errors Option” was set at “Constant Variance”. There has been no research on the effect of setting maximum joinpoints on the analysis results and but a number of studies have utilized three as the maximum Joinpoints [44–46]. Therefore, we chose the maximum number of joinpoints as 3 for the convenience when analyzing and interpreting data. Log transformation was used for all Joinpoint analyses. In the Advance tab, the “Grid search” method was selected and “Permutation Test” used to determine the best number of change-points in segmented line regression. The remaining parameters were set as default. (Fig 1)

The analyses were also conducted using SPSS version 22.0 (SPSS Inc, Chicago, IL, USA). The software was utilized to perform a descriptive statistics of productivity of each method in the EBM hierarchy [47,48]. An assessment of the normality of these data was retrieved by using the software feature. SPSS allows calculation of the maximum and minimum publication number of every article type with the corresponding years, the mean and standard deviation (SD), or the median and interquartile range (IQR).

In this study, the main parameter Annual Percent Change (APC) was used to describe trends. The APC was used to measure trends in medical research [40,49–52]. The APC from year t to year (t+1) can be acquired using the following formula:

$$APC\;\left(\%\right)=\frac{R_{\left(t+1\right)}-R_{t}}{R_{t}}\times 100=\left(e^{\alpha }-1\right)\times 100$$

Where R_t_ is the rate in year t and α is the slope coefficient in the linear equation below:

$$\text{ln}\left(R_{t}\right)=\alpha t+\beta$$

When describing trends over a fixed pre-specified interval, a *p-value* ≤ 0.05 was considered statistically significant [53,54].

## Results

**Fig 2:**
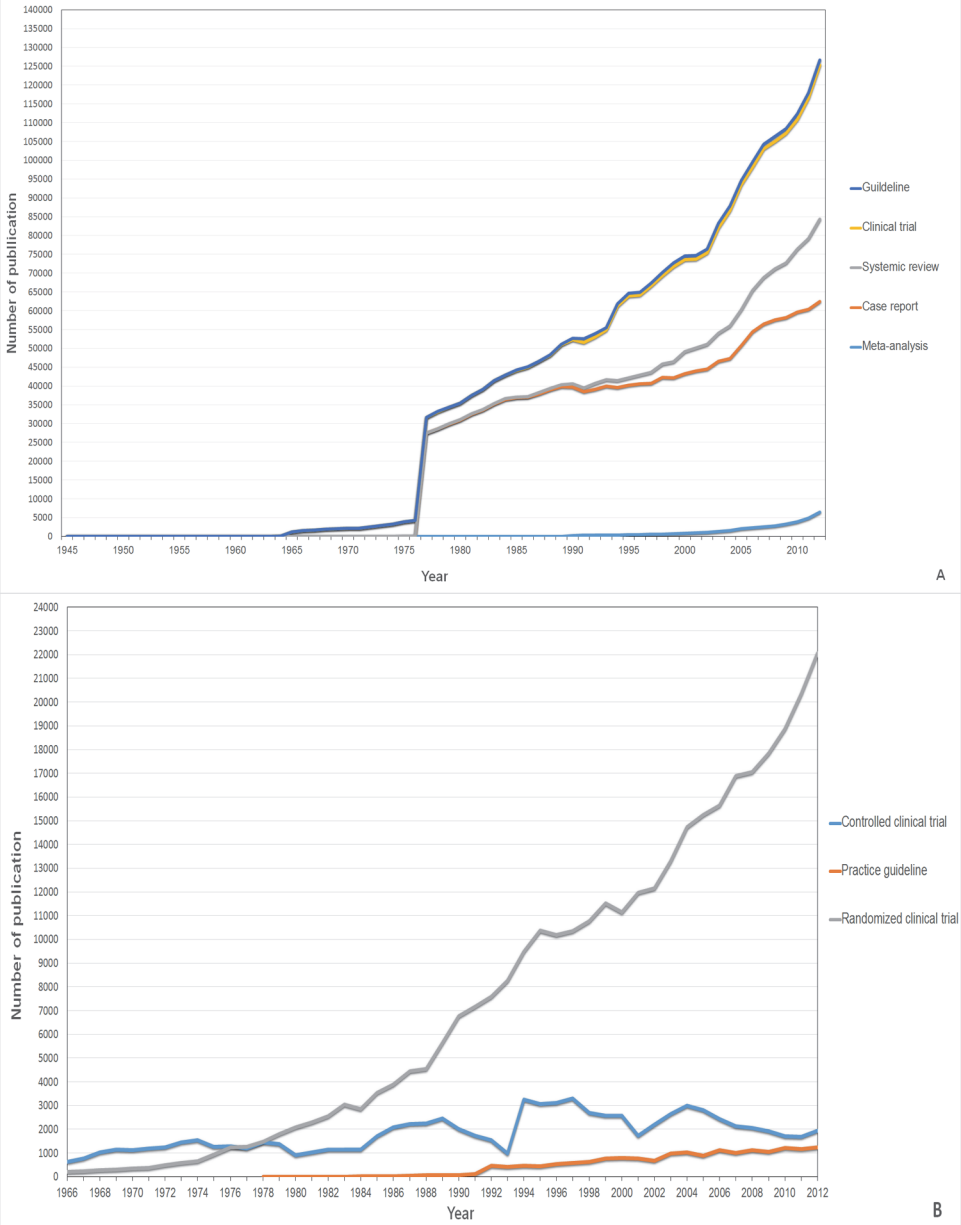
(A) Stacked area chart displaying the order of appearance and the trends of development of CR, CT, GL, SRV and MA regarding number of publications in PubMed over some time periods. (B) Stacked area chart displaying the order of appearance and the trends of development of RCT, CCT and PGL regarding number of publications in PubMed over some time periods

From the PubMed database, a total of 22,134,520 publications were extracted from the years 1945–2012. PGL and CR accounted for 0.08% and 6.75%, respectively, of APP, and comprised the smallest and largest categories of publication. The most recent year examined (2012) had the highest annual number of publications in each article type, except for CCT, which peaked in 1997. Among all publication types, PGL accounted for the least number of total papers (17,673 papers). RCT, SRV and MA accounted for a relatively small number of total publications (344,714 for RCT; 178,155 for SRV and 38,167 for MA). Non-RCTs accounted for 364,315 papers. (Table 1) (Fig 2).

**Table 1:** Evidence-based Medicine publications 1945–2012.

**Method**			**Year range**	**Total publication**	**Max (year)**	**Min (year)**	**Median**	^**+**^ **IQR**
All Pubmed publication			1945–2012	22134520	926293 (2012)	20242 (1945)	275347	330184.5
	**Case report**		1977–2012	1494328	55927 (2012)	27430 (1977)	39803.5	8713.8
	**Clinical Trial**		1961–2012	709029	40890 (2012)	1 (1962)	8095	21149.5
		**Controlled clinical trial**	1966–2012	85652	3302 (1997)	616 (1966)	1700	1244
		**Randomized controlled trial**	1966–2012	344714	22058 (2012)	194(1966)	5647	10725
	**Guideline**		1973–2012	23590	1398 (2012)	1 (1973,1977)	646.5	1091
		**Practice guideline**	1978–2012	17673	1235 (2012)	1 (1978)	452	951
	**Systematic review**		1945–2012	178155	21968 (2012)	1 (1945)	86.5	2237.3
	**Meta-analysis**		1990–2012	38617	6500 (2012)	272 (1990)	948	2168

^+^IQR: **Interquartile Range**

The timeline of APC for APP presented with an initial period of striking annual increase (APC = 75.3%, p<0.05), followed by a more sustained long period of more moderate increase (APC = 3.0–4.8%, p<0.05) (Table 2). The APC of CR-type publications showed four periods, in which three had continuous upward trends (APC = 4.3%, 0.8% and 4.8% in 1977–1984, 1984–2002 and 2002–2007, respectively, p<0.05) when one remained steady (APC = 0.8% in 2007–2012 p>0.05). Other types of article manifested increasing trends in number of papers during the recorded period. The exception was CCT, which had no significant change in the most recent fourteen years (APC = -2.56%, p>0.05). SRV and MA had continuous upward trends in the number of publications (APC of SRV = 3.3%, 23.6% and 12.2% in 1945–1970, 1970–1999 and 1999–2012, respectively; and APC of MA = 8.2% in 1990–1996 and 16.9% in 1996–2012, p<0.05).

**Table 2:** JoinPoint regression analysis of different APC trends.

**Method**			**Trend 1**		**Trend 2**		**Trend 3**		**Trend 4**	
			Years	^j^APC	Years	^j^APC	Years	^j^APC	Years	^j^APC
All Pubmed publication			1945–1947	75.3*	1947–1971	4.8*	1971–2002	3.0*	2002–2012	5.1*
	**Case report**		1977–1984	4.3*	1984–2002	0.8*	2002–2007	4.8*	2007–2012	0.8
	**Case report**/ **All Pubmed publication**		1977–1982	2.1*	1982–2004	-1.9*	2004–2007	0.7	2007–2012	-4.4*
	**Clinical Trial**		1961–1963	98.1*	1963–1966	566.2*	1966–2012	7.4*		
	**Clinical Trial**/ **All Pubmed publication**		1961–1963	79.1*	1963–1966	514.9*	1966–2012	4.0*		
		**Controlled clinical trial**	1966–1998	3.6*	1998–2012	-2.6				
		**Controlled clinical trial**/ **All Pubmed publication**	1966–1999	0.5	1999–2012	-7.1*				
		**Controlled clinical trial**/ **Clinical Trial**	1966–2012	-5.1*						
		**Randomized controlled trial**	1966–1973	15.8*	1973–1976	31.0*	1976–1992	12.6*	1992–2012	4.9*
		**Randomized controlled trial**/ **All Pubmed publication**	1966–1971	11.1*	1971–1979	18.3*	1979–1995	7.8*	1995–2012	0.2
		**Randomized controlled trial**/ **Clinical Trial**	1966–1981	9.8*	1981–1992	2.7*	1992–1996	-7.1	1996–2012	1.1*
	**Guideline**		1973–1983	11.2*	1983–1991	84.0*	1991–2012	4.2*		
	**Guideline**/ **All Pubmed publication**		1973–1983	7.8	1983–1991	79.0*	1991–2012	0.4		
		**Practice guideline**	1978–1994	48.6*	1994–2012	5.6*				
		**Practice guideline**/ **All Pubmed publication**	1978–1994	44.4*	1994–2012	1.5				
		**Practice guideline**/ **Guideline**	1978–1987	9.1*	1987–1991	-34.4*	1991–1994	63.2*	1994–2012	0.6
	**Systematic review**		1945–1970	3.3*	1970–1999	23.6*	1999–2012	12.2*		
	**Systematic review**/ **All Pubmed publication**		1945–1970	-2.5*	1970–1999	20.2*	1999–2012	7.1*		
	**Meta-analysis**		1990–1996	8.2*	1996–2012	16.9*				
	**Meta-analysis**/ **All Pubmed publication**		1990–1995	5.9*	1995–2012	11.7*				

_j_APC **= Annual percent changes calculated by JoinpointRegression Analysis**

*APC **is significantly different from zero when P < 0.05**

The proportion of CT, SRV and MA publications to APP publications has been increasing significantly during the most recent joinpoint period (APC of CT/APP, SRV/APP and MA/APP is 4.0%, 7.1% and 11.7%, respectively, with p<0.05). The CCT/APP and CR/APP proportion showed a downward trend in 1999–2012 (CCT/APP, APC = -7.1%, p<0.05) and 2007–2012 (CR/APP, APC = -4.4%, p<0.05) while other proportions including RCT/APP, GL/APP, PGL/APP show no significant change (p>0.05).

The proportion of CCT to CT had been exhibiting a gradually declining trend during the observed period from 1966 to 2012 (APC = -5.1%, p<0.05). The proportion of RCT to CT showed a different pattern: the number of publications rose continuously in 1966–1992 as well as in 1996–2012 (period 1966–1981: APC = 9.8%; 1981–1992: APC = 2.7% and 1996–2012: APC = 1.1%, p<0.05) while in 1992–1996 declined, but fell short of statistical significance (APC = -7.07%, p>0.05). The proportion of PGL/GL showed rising trends during two time periods (1978–1987: APC = 9.1%, p<0.05; and 1991–1994: APC = 63.2%, p<0.05) with a downward trend during the intervening period (1987–1991, APC = -34.4%, p<0.05). In recent years, the PGL/GL proportion has remained unchanged (APC = 0.6%, p>0.05).

## Discussion

This study demonstrates that article types with higher levels of evidence accounted for fewer publications than those with lower levels of evidence. One possible reason for this observation is that systematic review (SRV), randomized controlled trial (RCT), and meta-analysis (MA) articles may take longer to publish because of the time needed for establishing study design, data collection and analysis, as well as for peer review [55–57]. These article types also need more financial, technical and human resources to conduct. Data for SRV and MA are collected from available clinical trials, cohort studies, case-control studies, observational studies, etc. [55,56]. Additionally SRVs and MAs can only occur after a sufficient body of research has been undertaken which may take years if not decades. As a result of their inherent design, SRV and MA will mostly be produced at a much lower rate than other publication types which are ranked as having a lower quality of evidence. Similar to the case of SRV, RCT and MA, publishing practice guideline (PGL) is also time-consuming because of the time it takes to manage conflicts of interest, to assess the quality of evidence and to facilitate the consensus [58]. Case reports (CR) are ranked as being of the lowest quality of evidence but are nonetheless important for generating hypotheses for further studies to resolve new issues [37,59,60].

The overall slightly positive value of annual percent change (APC) for SRV and the negative APC in the proportion of SRV to All PubMed publication (APP) suggested that SRV has had a long period of slow growth and a decrease in popularity in the scientific world. However, since 1970, the APCs for SRV and the APCs in the proportion SRV/APP have increased dramatically. This increment might reflect a regain of interest in SRV among scientists. This may have been due to the reconsideration of the role of SRV as a critical contributor to Evidence-based Medicine (EBM) [61,62].

The marked increase in the APCs for MA, as well as the APCs for the proportion of MA to APP, suggests that MA is a popular methodology for publication in modern EBM. As a subset of SRV, MA uses a method that aims at combining results from several studies mathematically and hence create a larger sample size [55,56]. Combining data helps researchers assess more accurately the strength of relationship of two variables, enhances the statistical power of analysis, and narrows down the confidence interval. MA first appeared as recently as 1990, and have offered a number of advantages, including establishing a new benchmark in the quality of evidence.

For PGL, the growth was initially very strong (APC = 48.6%), but for the most recent 20 years, the growth rate has been slow (APC = 5.6%). As mentioned above, the recorded period for PGL was relatively short, and PGL currently has the lowest total number of publications. In the initial phase of growth, PGL appeared with a very small number of publications. Many questions have been raised about the quality of PGL. Initially some PGLs were assembled on consensus of expert committees rather than based on research derived evidence [63–66]. Clinical application of PGL was also an emerging problem for PGL makers [64,65,67]. The establishment of standardized criteria for developing PGL, beginning with the criteria of the Institute of Medicine (IOM) in 1990, opened a new era in which more focus was placed on strengthening recommendations through strong evidence, and translating evidence into concise instructions for clinical practice [68]. This fact may explain why the growth rate of PGL has decreased over the past 20 years.

This study found a slight increase in growth in the number of published clinical trials (CTs) ((APC = 7.4% for CT). As mentioned earlier, CT is still important for generating hypothesis and creating data for further in-depth studies, namely SRV and MA. We hypothesize that as APP continues to grow in number, the aforementioned article types will continue to increase, as the scientific community will continue to face new issues and open up to new perspectives.

RCTs are more prevalent than controlled clinical trial (CCTs), as shown by their respective total absolute quantities (Table 1), the stable APCs in the proportion RCT/CT and the reduction in the APCs in CCT/CT (Table 2). Methods used in RCT offers advantages such as a reducing effect on biases and errors as well as the establishment of a strong statistical power, and enable scientists to obtain high-quality evidence and valuable data for SRVs and MAs [69]. Nowadays, RCT is used extensively in most Phase III and some phase II clinical trials [69,70]. CT had very large APCs in the 1960’s. This marked increase may be explained by significant changes in the definition of CT during the 1950s [71]. After this period, plenty of treatment methods were invented and proved effectiveness by using CT method. Besides, the promotion for CT, which has been seen in that era as the gold standard for clinical studies, the most eloquent proof for the treatment effectiveness, had somewhat supported the rapid growth in the number of publications in this article type.

The aim of this study was to assess the tendency of changes in quantities of publication types which are used in evidence-based medicine, and thus to partly show researchers’ interest in various publication types. We considered some publication types such as MA, SRV, RCT as providing high level of evidence; however, according to OCEBM 2011 [34], the level of evidence may be downgraded because of study quality, impreciseness, indirectness, too small absolute sample size or inconsistency between studies. Further studies are required to analyze the effect of publication types on EBM, especially with qualitative approaches. The current study utilized data derived from the Medical Subject Headings (MeSH) system of PubMed; therefore, available studies which are not included in MeSH are not included in our analysis. Some of the year ranges were not significant maybe because of the short examined regression segment.

## Conclusion

In conclusion, quantitative growth was found across all publication types. The growth of all, rather than only some publication types, is necessary for the development of EBM, because evidence-based medicine develops in a step-by-step manner: evidence from low-quality studies serve as data points for conducting larger/better designed studies which provide stronger evidence. Current trends may predict that SRV and MA will continue to grow in the future. RCT is getting priority over the other subset of CT; and although the proportion of RCT to CT has recently shown a stable but significantly increasing trend, RCT is still important in order to gain valuable robust evidence needed for better health care outcomes. This current study provides the first large scale quantitative analysis of EBM publication trends.
